# Hydrogen Peroxide and Sodium Transport in the Lung and Kidney

**DOI:** 10.1155/2016/9512807

**Published:** 2016-03-17

**Authors:** V. Shlyonsky, A. Boom, F. Mies

**Affiliations:** ^1^Laboratoire de Physiologie et Pharmacologie, Université Libre de Bruxelles, 1070 Bruxelles, Belgium; ^2^Laboratoire d'Histologie, Histopathologie et Neuroanatomie, Université Libre de Bruxelles, 1070 Bruxelles, Belgium

## Abstract

Renal and lung epithelial cells are exposed to some significant concentrations of H_2_O_2_. In urine it may reach 100 *μ*M, while in the epithelial lining fluid in the lung it is estimated to be in micromolar to tens-micromolar range. Hydrogen peroxide has a stimulatory action on the epithelial sodium channel (ENaC) single-channel activity. It also increases stability of the channel at the membrane and slows down the transcription of the ENaC subunits. The expression and the activity of the channel may be inhibited in some other, likely higher, oxidative states of the cell. This review discusses the role and the origin of H_2_O_2_ in the lung and kidney. Concentration-dependent effects of hydrogen peroxide on ENaC and the mechanisms of its action have been summarized. This review also describes outlooks for future investigations linking oxidative stress, epithelial sodium transport, and lung and kidney function.

## 1. Introduction

It is generally accepted that cells in most tissues are exposed to some level of H_2_O_2_ and locally this level may reach significantly high concentrations. Multiple studies have reported that high levels of H_2_O_2_ (usually >100 *μ*M) are cytotoxic to a wide range of animal, plant, and bacterial cells in culture, although LD_50_ values depend on the cell type, length of exposure, and the cell culture conditions [1–5]. It is therefore widely thought that H_2_O_2_ is very toxic* in vivo* and must be rapidly eliminated. It is, however, paradoxical that patients with acatalasemia (autosomal recessive peroxisomal disorder caused by a complete lack of catalase that neutralizes H_2_O_2_) rarely have health problems [1]. Knockout of glutathione peroxidase, enzyme that also reduces free hydrogen peroxide to water, does not induce any particular phenotype [6, 7]. This suggests that hydrogen peroxide besides participation in innate immunity may have also important signaling and/or regulatory role in living organisms [8].

Ion transport by the lung epithelial cells is the major mechanism that maintains optimal level of lining airway and alveolar liquid. This in turn determines efficient aeration of the lung and facilitates diffusion of gases across the alveolar-capillary walls. In the kidney, sodium reabsorption in different parts of nephron regulates fluid balance and thus blood pressure. Therefore, intracellular signaling cascades that regulate ion transport mediated by ion channels are of particular interest for any fundamental and clinical investigation of the lung and kidney function. The role of reactive oxygen species [ROS] in modulation of ion channels activity has been recently recognized. Ion channels regulation by these reactive species may occur in several different ways [9]. First way is through direct oxidation of key amino acid residues of channel proteins. Second, reactive species may alter the activity of other signaling mechanisms that secondarily lead to changes in channel activity or channel gene expression. At last, there are more complex mechanisms mediated through alterations in trafficking or turnover of channel proteins through changes in proteasomal degradation of channels (rew in [9]).

The purpose of this review is to explain the role and the origin of H_2_O_2_ in lung and kidney and its concentration-dependent effects on sodium transport, particularly on epithelial sodium channel (ENaC). This review also describes outlooks for future investigations linking oxidative stress, epithelial sodium transport, and lung and kidney function.

## 2. Sources of H_2_O_2_ and Estimation of* In Situ* Concentration

### 2.1. Lung

Reactive oxygen species (ROS) in the lung may have exogenous and endogenous origin. First, ROS may be present in inhaled air that contains cigarette smoke, environmental pollutants, and oxidant gases. Alternatively, hydroperoxides [e.g., H_2_O_2_], superoxide anions, and hydroxyl free radicals can be generated by activated inflammatory cells [such as neutrophils, eosinophils, and alveolar macrophages] and by epithelial and endothelial cells themselves [10]. These cellular ROS are formed as intermediates of the incomplete reduction of oxygen in mitochondrial electron-transport systems, by microsomal metabolism of endogenous compounds and xenobiotics, or by various enzymatic generators, such as xanthine oxidase. Endothelial, inflammatory cells and pneumocytes generate and release ROS via an NADPH oxidase-dependent mechanism, which is mediated by membrane receptor activation of phospholipase C leading to elevation of intracellular calcium level [11]. Finally, there is convincing data suggesting that hydrogen peroxide, produced in small intestine, enters mesenteric lymph and finds its way to the lung [12].

Production of H_2_O_2_ has been detected both in alveolar cell cultures* in vitro* [13–16] and in the exhaled breath of humans [17, 18]. ROS in exhaled breath condensate (EBC) have been measured in different inflammatory lung diseases (asthma, chronic obstructive pulmonary disease, cystic fibrosis, etc.) with majority of the clinical reports showing increased ROS concentration in patients compared with normal subjects [17, 18]. Lack of or very weak correlation of ROS levels in EBC with other biological fluids such as bronchoalveolar lavage liquid and sputum have been also reported [19]. High variability of the measurements for the same subjects was also observed and levels of H_2_O_2_ showed correlation with circadian rhythms and the diet. Maximal concentration of hydrogen peroxide, the most abundant ROS species in the lung, does not exceed 0.9 *μ*moles per liter of EBC obtained from normal subjects [17–19]. The major difficulty consists in translation of this value into the concentration of H_2_O_2_ in alveolar lining fluid, that is,* in situ* concentration. Hydrogen peroxide has much lower volatility as compared to water and there exists exponential relationship between the molar H_2_O_2_/H_2_O fraction in the solution and in the vapor phase [20]. However, since micromolar concentration of H_2_O_2_ in EBC gives molar ratio in vapor phase of 1.8*∗*10^−8^, it is impossible to extrapolate the existing data on molar fractions in liquid phase to such a low value.

Alternative way to estimate absolute H_2_O_2_ concentration* in situ* is to measure peroxide concentration in bronchoalveolar lavage fluid (BALF) and correct the value by a factor obtained by urea dilution technique [21]; however, no research groups have done it so far. Nevertheless, there are published reports with separate data on hydrogen peroxide concentration in BALF and on alveolar lining fluid volume. In normal human lungs, alveolar lining fluid volume fluctuates within the range of 0.2–2.0 mL [22–26], while in rats this volume has been found to be within the range of 0.03–0.26 mL [27–29]. Since commonly used volume of liquid for bronchoalveolar lavage in humans is 100 mL and 5 mL in rats, the expected dilution factor is likely to be ~100 in normal lungs [30]. Knowing that the concentration of H_2_O_2_ in BALF is 0.14–0.70 *μ*M in rats [31, 32] and 0.08 *μ*M in humans [33], actual hydrogen peroxide concentrations in the lungs* in situ* may be well expected in micromolar to tens-micromolar range. However, these are just approximations and they warrant further studies.

### 2.2. Kidney, Urinary Tract, and Bladder

Freshly voided human urine may contain substantial quantities of H_2_O_2_, at concentrations sometimes exceeding 100 *μ*M [34–36]. The H_2_O_2_ detected in human urine appears to arise, at least in part, by O_2_^∙−^-dependent autoxidation of urinary molecules [36, 37]. H_2_O_2_ can be generated by activated phagocytes in urine and can be generated in the kidney by NADPH oxidases. Indeed, reactive oxygen species are produced by fibroblasts, endothelial cells, vascular smooth muscle cells, mesangial cells, tubular cells, and podocytes cells [38]. NOX-1 and NOX-4 are expressed in the kidney, with a prominent expression in renal vessels, glomeruli, and podocytes, and cells of the thick ascending limb of the loop of Henle, macula densa, distal tubules, collecting ducts, and cortical interstitial fibroblasts [38]. NADPH oxidase activity is upregulated by prolonged infusion of angiotensin II or by a high salt diet [38]. The high levels of H_2_O_2_ that can be detected in some urine samples strongly suggest that at least some H_2_O_2_ generation occurs within the bladder. Urinary levels of H_2_O_2_ can also be increased by diet supplement (e.g., coffee drinking), by enzymatic reaction directly in urine, and by general oxidative stress. This suggests possibility that excretion of H_2_O_2_ represents a metabolic mechanism for controlling its levels in the human body. Accordingly, a measurement of urinary H_2_O_2_ levels represents a valuable tool for assessment of oxidative stress, since H_2_O_2_ can be quantified very fast and with high precision.

### 2.3. Blood

Hydrogen peroxide observed in the lung and the kidney might finally find its way from the blood. Some studies have claimed substantial levels of H_2_O_2_ (up to ~35 *μ*M) in human blood plasma [39–41]. However, these studies have been performed under assay conditions in which removal of H_2_O_2_ was prevented. This implies that human plasma may continuously generate H_2_O_2_. One enzyme involved in this process, at least under pathological conditions, appears to be xanthine oxidase [42]. In the plasma, H_2_O_2_ can react with heme proteins, ascorbate, and protein-SH groups and it is degraded by the traces of catalase present, and thus, under conditions of keeping normal antioxidant mechanisms, levels of H_2_O_2_ are reported to be very low, close to zero [43].

## 3. Regulation of Sodium Channels by Hydrogen Peroxide

Recent studies suggest that the expression and the activity of ENaC may be regulated by the oxidative state of the cell. Direct application of 100 *μ*M H_2_O_2_ to A6 distal nephron cells upregulates ENaC open probability and equivalent transepithelial sodium open-circuit current [44–47]. Furthermore, pharmacological inhibition of NADPH oxidase NOX-4 by fulvene-5 in A6 cells downregulates ENaC activity [47]. Thus, this suggests that tonical production of H_2_O_2_ by NOX-4 supports single-channel activity of ENaC. Similar stimulatory effect of exogenous H_2_O_2_ at 250 *μ*M has been obtained by Downs and colleagues in lung epithelial cells [48]. They have shown that single-channel activity of both highly selective and nonselective ENaCs is upregulated in type 2 pneumocytes [48]. In our experiments on formation of epithelial domes on nonporous support, which is reflective of ENaC activity [49], catalase and superoxide scavenger TEMPO inhibited up to 80% of dexamethasone-induced dome formation in H441 lung epithelial cells and submillimolar concentrations of H_2_O_2_ could alone transiently stimulate this dome formation [our unpublished data].

Another reactive oxygen species, superoxide anions O_2_^∙−^ produced by NOX-2, has been shown to positively regulate ENaC activity in alveolar cells [50]. Superoxide anion may have also indirect stimulatory action on ENaC single-channel activity by neutralizing NO that decreases ENaC activity [51]. In addition, agents that increase local superoxide concentration (mixture of hypoxanthine compounds and xanthine oxidase) stimulate single-channel activity of ENaC in A6 epithelial cells [52].

At the level of protein expression, exogenous hydrogen peroxide has been shown to inactivate ubiquitination of lung *α*-ENaC, thus increasing its surface expression [48]. But at the level of gene expression, contrasting effects of H_2_O_2_ on *α*-, *β*-, and *γ*-ENaC have been reported [48, 53, 54]. While low doses below 0.25 mM have no significant effect on transcription [48], near millimolar concentrations of H_2_O_2_ suppress *α*-ENaC transcription [53, 54]. In type II pneumocytes, cyclic AMP and activation of glucocorticoid receptors stimulate the expression and activity of ENaCs as well as the expression of H_2_O_2_ producing NADPH oxidase DUOX1 [15, 55], which leaves the possibility for a speculation that long-term activation of single-channel activity of ENaC by cAMP and dexamethasone might be in part related to H_2_O_2_ production by DUOX1.

Molecular mechanisms of ENaC activity stimulation by H_2_O_2_ seem to involve activation of PI3-kinases that produce anionic phospholipids phosphatidylinositol-4,5-bisphosphate, PIP2, and phosphatidylinositol-3,4,5-trisphosphate, PIP3 [44–46]. In addition to activation of PI3 kinase, increase in PIP3 in A6 cells in the presence of H_2_O_2_ may be explained at least in part by inactivation of PTEN that negatively regulates intracellular levels of PIP3 [56, 57]. PIP2 and PIP3 in turn bind to ENaC or participate in other signaling cascades and by doing so modulate cellular sodium transport (rew in [58]). In this regard it has been shown that epidermal growth factor (EGF), insulin, insulin growth factor-1 (IGF-1), and prorenin have a common stimulatory effect on ENaC in renal cells that is mediated by ROS production and hydrogen peroxide in particular [59, 60]. In lung epithelial cells, hydrogen peroxide produced by DUOX1 extracellularly may reenter the cell, where it activates PI3-kinase, which in turn stimulates ENaC activity. Catalase action suggests that hydrogen peroxide has no feedback effect on DUOX1 gene expression, while there is possibility that it may negatively regulate ENaC transcription [42, our unpublished observations]. Taken together, stimulation of ion transport activity of ENaC and its stabilization at the cell surface by decreasing ubiquitination by H_2_O_2_ might be balanced at least in the lung by negative feedback of hydrogen peroxide on the ENaC transcription.

The above stimulatory action of hydrogen peroxide on ENaCs contrasts the reports obtained in studies with severe oxidant stress on lung epithelial cells, in which downregulation of sodium transport has been reported. In lung epithelial cell monolayer studies, severe oxidative stress induced by millimolar concentrations of H_2_O_2_ alters epithelial ion transport mechanisms by decreasing short-circuit current (*I*_sc_) and monolayer resistance (*R*), while being more effective from the basolateral (serosal) side [61]. In this study, the effective concentration of apical H_2_O_2_ at which *I*_sc_ was decreased by 50% was absolutely nonphysiological and equal to 4 mM [61]. It has been also reported that exogenous hydrogen peroxide in excess of 200 *μ*M interferes with glucocorticoid-induced transcription of *α*-ENaC subunit in A549, H441, and Calu-3 lung epithelial cells [53, 54].

Another way to create a condition of severe oxidative stress consists in application of significant concentration of glutathione disulfide (GSSG). Zhang and colleagues have reported nanomolar concentrations of GSSG in one milliliter of medium obtained after lysis of 2*∗*10^5^ HL60 cells [62]. For a cell of 10 *μ*m in diameter having cellular volume of approximately 0.5 pL, this corresponds to a 10^4^ dilution factor and thus only micromolar to ten-micromolar intracellular concentrations of GSSG can be expected in resting cells. Downs and colleagues have shown that direct application to lung epithelial cells of 400 *μ*M GSSG induced decrease in ENaC open probability [63]. This GSSG concentration is at least ten times higher than in resting cell and clearly represents a condition of severe oxidative stress. The inhibitory effect of GSSG was explained by the reversible formation of mixed disulfides between glutathione and low-pKa cysteinyl residues of ENaC and possibly by irreversible oxidation of the latter [63].

## 4. Perspectives and Outlooks

Epithelial sodium channel ENaC in the distal nephron is the major ion channel responsible of maintaining Na homeostasis by fine-tuning of Na reabsorption, thus playing an important role in the long-term control of arterial pressure. Excess of ENaC activity leads to systemic hypertension. In the kidney, ENaC is expressed in cortical collecting duct cells (CCD); these cells are able to produce and secrete ROS, particularly hydrogen peroxide [64] which may lead to a stimulation of sodium transport [46]. H_2_O_2_ is very diffusible within and between the cells and accordingly H_2_O_2_ generated in kidney could play a role in sodium reabsorption. ROS participate in the regulation of ENaC and other channels and transporters in the CCD and thus their generation might be linked to diseases associated with ionic channels. It has been shown in several experimental models of hypertension [65] and hypertensive patients [65–67] that ROS levels are increased. In chronic renal insufficiency, H_2_O_2_ levels* in situ* are increased as well [64]. Moreover, high salt intake can elevate both superoxide [O_2_^∙−^] [68–70] and H_2_O_2_ in the kidney by stimulating NADPH oxidase [71, 72] and thus furthering sodium retention and aggravating systemic hypertension through modulation of ENaC activity. Moreover, urinary excretion rate of hydrogen peroxide is closely related to metabolism of electrolytes and fluid in the renal tubules [35]. Therefore, it seems that reactive oxygen species play a pathophysiological role in the development of essential hypertension and this may implicate their action on ENaCs [73, 74].

Dietary polyphenols from green tea and red vine are well known as blood pressure lowering agents and many studies show an inverse correlation between dietary consumption of these polyphenols and reduced incidence and mortality from cardiovascular diseases [75–77]. A reduction in the markers of oxidative stress induced by dietary polyphenols in different animal models of hypertension could be a mechanism involved in the blood pressure lowering effect [78, 79]. One cannot exclude direct renal effects in the antihypertensive action of polyphenols, since, for instance, the downregulation of ENaC in the kidney by flavonoid quercetin contributed to the blood pressure lowering effect in Dahl salt-sensitive hypertension [80, 81].

ENaC is also major player that participates in fluid absorption in the lung. In cystic fibrosis [CF], the absence of functional cystic fibrosis transmembrane regulator channel (CFTR) upregulates the ENaC channel activity and further decreases salt and water secretion by reabsorbing sodium ions. On the other hand, cystic fibrosis lung disease is characterized by chronic airway inflammation and thus oxidative stress, which can be quantified by measurement of hydrogen peroxide in exhaled breath condensate [17, 82]. It is then plausible that increased production of H_2_O_2_ in CF lungs contributes to sodium hyperabsorption. This suggestion merits an investigation of the link between these two phenomena. In this regard it has been shown that oxidation arising from airway inflammation or environmental exposure contributes to pathologic mucus gel formation in the lung and such antioxidants as polyphenol resveratrol and N-acetyl-cysteine have clear mucolytic activity [83, 84]. It would be also interesting to see the effect of nebulized catalase on mucociliary clearance in healthy and CF subjects or in animal models, although it may compromise innate immunity in the lungs.

## 5. Conclusion

Review of the literature suggests that hydrogen peroxide has a stimulatory action on ENaC single-channel activity and on its stability at the membrane while having possible inhibitory action on the transcription of the ENaC subunits. Under conditions of severe oxidative stress of the cell, this channel activity is inhibited. Modulation of ENaC activity by H_2_O_2_ might contribute to the development of such pathophysiological conditions as systemic hypertension and thickening of the mucus in the CF lungs, although no direct evidence in support of these hypotheses has been provided so far. As a concluding note for perspectives, we suggest that further studies on ENaCs may focus on the action of dietary polyphenols on the activity and expression of this channel in lung and renal epithelial cells. These studies should be performed in conjunction with the measurements of oxidative state of these cells including* in situ* measurements of absolute values of hydrogen peroxide concentrations.
